# Substrate-Related Factors Affecting Cellulosome-Induced Hydrolysis for Lignocellulose Valorization

**DOI:** 10.3390/ijms20133354

**Published:** 2019-07-08

**Authors:** Ying Wang, Ling Leng, Md Khairul Islam, Fanghua Liu, Carol Sze Ki Lin, Shao-Yuan Leu

**Affiliations:** 1 Guangdong Key Laboratory of Integrated Agro-environmental Pollution Control and Management, Guangdong Institute of Eco-Environmental Science & Technology, Guangzhou 510650, China; 2 Department of Civil and Environmental Engineering, the Hong Kong Polytechnic University, Hung Hom, Kowloon, Hong Kong SAR, China; 3 Bioproduction Research Institute, National Institute of Advanced Industrial Science and Technology (AIST), Central 6, Higashi 1-1-1, Tsukuba, Ibaraki 305-8566, Japan; 4 School of Energy and Environment, City University of Hong Kong, Tat Chee Avenue, Kowloon, Hong Kong SAR, China

**Keywords:** cellulosome, lignocellulose, substrate-related factors, enzymatic hydrolysis, bioproducts, reaction kinetics

## Abstract

Cellulosomes are an extracellular supramolecular multienzyme complex that can efficiently degrade cellulose and hemicelluloses in plant cell walls. The structural and unique subunit arrangement of cellulosomes can promote its adhesion to the insoluble substrates, thus providing individual microbial cells with a direct competence in the utilization of cellulosic biomass. Significant progress has been achieved in revealing the structures and functions of cellulosomes, but a knowledge gap still exists in understanding the interaction between cellulosome and lignocellulosic substrate for those derived from biorefinery pretreatment of agricultural crops. The cellulosomic saccharification of lignocellulose is affected by various substrate-related physical and chemical factors, including native (untreated) wood lignin content, the extent of lignin and xylan removal by pretreatment, lignin structure, substrate size, and of course substrate pore surface area or substrate accessibility to cellulose. Herein, we summarize the cellulosome structure, substrate-related factors, and regulatory mechanisms in the host cells. We discuss the latest advances in specific strategies of cellulosome-induced hydrolysis, which can function in the reaction kinetics and the overall progress of biorefineries based on lignocellulosic feedstocks.

## 1. Introduction

Bioproducts, including biofuels and value-added chemicals derived from renewable resources, provide sustainable alternatives for petroleum-based products which contribute to climate change and energy crisis [1,2]. Among the variety of renewable resources, lignocellulosic biomass is the most abundant and economical carbon source on the earth. The development of bioproducts converted from lignocellulosic biomass should ultimately be essential for sustainable development without threatening food supplies and human survival [3]. However, as a natural protective barrier, the structure of plant cell wall is a recalcitrant network composed of cellulose, hemicellulose, and lignin, which is extremely difficult to degrade into fermentable sugars. Therefore, cellulose degradation and sugar release are becoming the typically rate-limiting factor for lignocellulosic biomass utilization [4]. Various efforts have been paid to gain access and deconstruct fermentable sugars in lignocellulosic biomass. In the existing biorefinery process, commercial exogenous cellulases are employed to hydrolyze lignocellulosic biomass synergistically, whereas the large amount of cellulase consumption would almost counteract its benefit of using low-cost feedstock [5]. Combining microbial enzyme generation, saccharification with fermentation in one-step, the consolidated bioprocessing (CBP) has been accepted as an economically feasible strategy for bioproduct conversion from lignocellulosic biomass [6]. Although some aerobic fungi such as *Trichoderma reesei*, *Aspergillus niger*, and *T. koningii* exhibit potential cellulase extracellular secretion by the common natural habitats of these microorganisms, the requirement of continuous oxygen supply and nutrient competition with other co-cultured microorganisms has limited the possibilities of CBP with fungi [7]. Recently, cellulosomes—the multienzyme complexes produced by certain anaerobic cellulolytic bacteria have gained considerable attention, owing to their specifically design to overcome the natural recalcitrant network consisted by plant cell wall polysaccharides [8,9]. It has been reported that the polycellulosomes are as large as 100 MDa in nature, and the cellulosomes range in mass is 650,000 Da–2.5 MDa [10]. Therefore, as one of the most efficient naturally occurring biocatalysts to degrade lignocellulosic biomass, cellulosomes are potential substitutes for reducing enzyme loading in industrial scale biorefineries.

**Figure 1:**
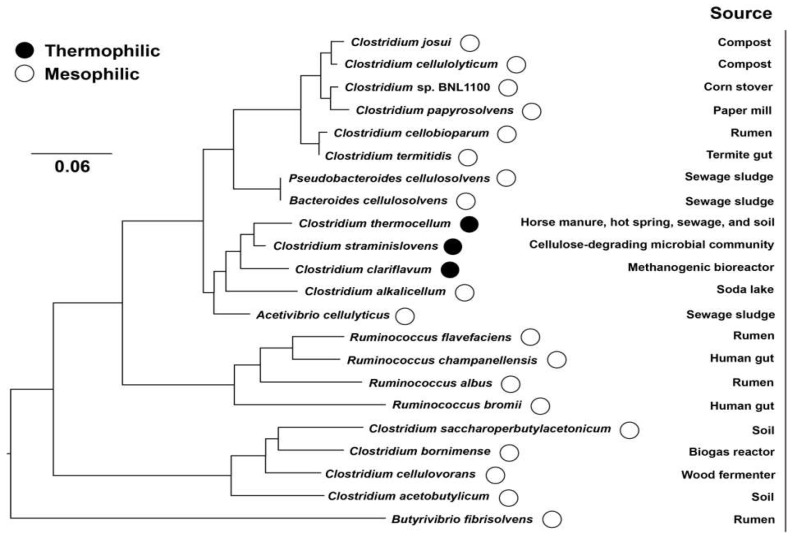
Phylogenetic tree based on 16S rRNA sequencing of the anaerobic cellulosome-producing bacteria.

The supermolecular cellulosome complexes were first described in the cellulolytic thermophilic *Clostridium thermocellum* in the early 1980s [11]. Generally, cellulosomes consist of non-enzymatic scaffolding proteins associated with a variety of enzymatic subunits that play a decisive role to degrade cellulose and hemicellulose. The architectures and components of the multienzyme systems are various with different bacteria [12]. The main functions of cellulosomes include: (i) improvement of substrate uptake; (ii) tighten the specific interaction with certain substrates; and (iii) synergistic activity and processivity of cellulases [13]. Interestingly, cellulosomes have been verified to degrade not only crystalline cellulose, but also non-crystalline hemicelluloses, or even chitin and pectin [14]. The major producers of cellulosomes can be classified into several genera, i.e., *Clostridium*, *Ruminococcus*, *Acetivibrio*, *Bacteroides*, and *Pseudobacteroides* belong to both mesophile and thermophile [5,8,10,15] (Figure 1), but no cellulosome has been identified in microorganism that can grow above 65 °C and in the Archaea. These microorganisms exist in various environmental niches, such as sewage sludge, soil, animal guts, rumen, and wood chip piles. The different microbial sources are constantly observed by characterization and comparison of the cellulosomal enzyme properties.

In recent times, numerous attempts have been described to improve cellulosomal catalysis by maximizing enzyme activities and/or creating the synergy between cellulosomal hydrolysis and the consequential fermentation [16,17]. Most of the efforts focus on engineering an ideal microorganism for CBP application, although the interaction between cellulosomes and lignocellulosic substrates remains to be clarified. This article presents a review of recent advances involved in properties of cellulosomes with respect of the composition and structural characterization. Moreover, the substrate-related physical and chemical factors affecting cellulosome adsorptions and catalytic activities are discussed in detail. We also describe the enzyme diversity and regulatory mechanisms of cellulosomes, and their latest achievements and limitations in potential CBP of lignocellulosic biomass to bioproducts. 

## 2. Cellulosome Composition and Assembly

**Figure 2:**
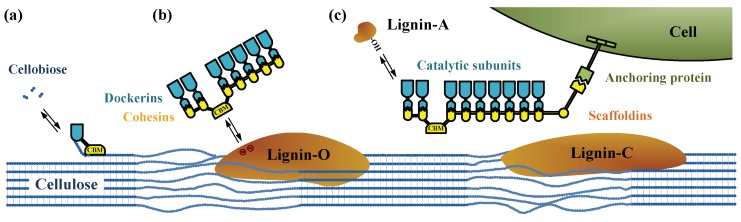
Cellulosomal assemblies and the hypothetical interactions with pretreated substrates. (**a**) free enzymes; (**b**) cell-free scaffodins; and (**c**) on cell wall. Lignin-O: sulfite treated; Lignin-A: dissolved; and Lignin-C: condensed lignin.

The mechanisms of cellulosome assemblies are one of the greatest interests to reveal the structure–function relationship. Efficient degradation of lignocellulosic biomass by cellulosomes requires appropriate composition of enzymes and optimal cellulosome structures. The estimated molecular mass of individual cellulosome produced by different microorganism ranges from 2 × 10^6^ to 6 × 10^6^ [18]. The cellulosome consists of two major components, namely (i) non-enzymatic scaffoldins including enzyme-binding sties named cohesins and carbohydrate-binding module (CBM); (ii) catalytic enzymes with dockerins interacting with cohesins in scaffoldins (Type I interaction) or surface layer homology domain (Type II interaction) [9,10]. Figure 2 shows the assembly of each component of cellulosomes on the cell surface and their possible interactions with lignocellulosic substrate derived after different types of pretreatment processes. These micro-structures can either suspended freely in the liquid (Figure 2a), connect to intermediate scaffodins (Figure 2b), or bind on the bacteria cell wall (Figure 2c). In this review, we summarize the cellulosome-related factors in Section 2 and the substrate-related factors in Section 3.

### 2.1. Scaffoldin

The structure of scaffoldin forms the backbone of the enzymatic subunits, which is assembled by the dockerins. The scaffolding proteins contain one or more cohesin domains (Coh) and binding to substrates via CBM [19]. There are mainly three types of scaffoldin, i.e., primary, anchoring, and adaptor scaffoldins. Among them, the primary scaffoldin is the most common one and contains numerous Cohs that interact with dockerin-containing enzymes [20,21]. Although the mechanism by which a single primary scaffoldin can attach to the cell surface remains unknown, it is deduced that the scaffoldin should play a regulatory role during the assembly of cellulosome by using different substrates [10,21]. 

### 2.2. Cohesin–Dockerin Interaction

The cohesin–dockerin interaction can be considered as a mechanism of plug-and-socket in which the cohesin socket is plugged by the dockerin [22]. The various sequences of cohesin and dockerin are associated with the signature sequences of the cellulosomal enzymes [23]. In other words, the heterogenous nature of cellulosomes caused by the interactive variability of cohesin–dockerin pairs with different expressions in cohesin repeats, enzyme connections to the scaffoldins and species-specific variations [24,25,26]. The cohesin–dockerin interaction is known as one of the strongest protein–protein interactions in nature, even approaching the strength between high-affinity antigen and antibody (Ka–10^11^ M^−1^) [27,28]. There are three types of cohesin–dockerin interaction have been reported according to sequence homologies of the cohesins and their binding partners, i.e., Type I, II, and III interactions [29,30]. Type I interactions are located between dockerin-containing enzymatic subunits and anchoring scaffoldins. Type II interactions are usually located between anchoring scaffoldins and enzyme-binding primary scaffoldins [9]. In addition, Type III interactions do not interact with either Type I or Type II domains [29]. Type I and Type II interactions are observed in *Clostridium* spp., while Type III interactions exist in ruminococcal cellulosomes [31].

### 2.3. CBMs (CBDs)

The cellulosomal CBM also called cellulose binding domain (CBD), belonging to carbohydrate-binding module family 3, is present on scaffoldins that bind the cellulosome tightly to the cellulosic substrate by disrupting its crystal surface at the solid–liquid interface [32,33]. Besides cellulose, some CBMs such as the CBM of *Clostridium cellulovorans* can also bind to chitin, which has similar crystalline structure to cellulose [34]. Although the CBM is a non-catalytic domain, it brings the cellulosomal enzymes close to its substrate, and therefore making the hydrolysis more efficient compared to free enzymes [35,36,37]. The CBM specific binding with substrate depends on the content and arrangement of amino acids [38]. For instance, cellulosomal CBM recognizes crystalline cellulose as reflected in homologous binding surface, which consists of mostly polar and aromatic side chains [39,40].

### 2.4. Cellulosomal Enzymes

The cellulosomal enzymes were first described in *Clostridium thermocellum* by cloning and expressing genomic libraries [41]. Cellulosomes usually exhibit better breakdown of substrates compared to free enzymes owing to their close proximity of the expressed enzymes, which act synergistically [42,43]. In general, the free enzymes depend on a CBM for guiding their catalytic domains to the substrates, whereas a dockerin domain located on the cellulosomal enzymes by which the enzymes are incorporated into the cellulosome complex. In this manner, cellulosomal enzymes contain the catalytic domains assembled by the duplicated dockerins linked to cohesins in scaffoldins via calcium dependent interactions [44,45]. 

So far, almost all cellulosome producers are characterized to produce large amount of glycoside hydrolase 48 (GH48) exoglucanase, which is crucial for enzymatic activity [46,47]. Intriguingly, not only cellulases but also hemicellulases [48,49,50,51] and other carbohydrate-active cellulosomal enzymes such as ligninases [52,53], pectinases [54,55], mannanases [56,57,58], and chitinases [59,60] were subsequently identified with the cellulosomes. These plant polysaccharide-degrading enzymes are highly complex and diverse, which makes it difficult to understand the mechanisms of protein assemblies and organizations. It has been reported that the complex cellulosomal architecture is responsible for minimizing the diffusion of certain carbohydrates and facilitate their uptakes by the cellulosomal enzymes for complete degradation [9,61]. The expressions and activities of cellulosomal catalytic subunits can be varied according to the substrate availablity [62,63,64]. Cellulosomes with different compositions can be assembled on a microorganism with various enzyme complexes when grew on different carbon sources [65]. Therefore, understanding of the interactions between cellulosomes and the utilized substrates is crucial to reveal the mechanisms of cellulosome expression and apply the potential CBP for bioproducts conversion.

## 3. Effects of Several Substrate-Related Factors on Cellulosome-Induced Hydrolysis

**Figure 3:**
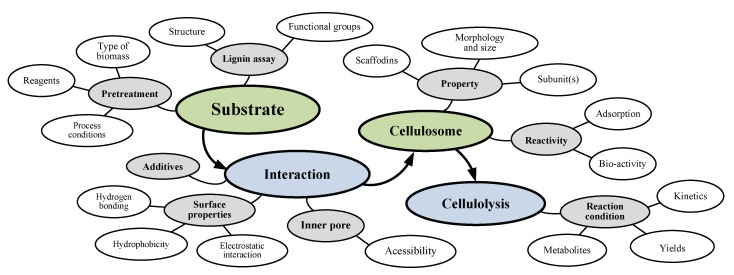
Interactions between cellulosome and substrate-related factors.

Despite the cellulosome complex being certainly viewed as an efficient natural system to break down the lignocellulosic biomass, there are several substrate-related factors that determine the reaction kinetics and yield of degradation of plant polysaccharide to its respective short chain monomers. These include: (i) carbon sources, i.e., types and sizes of lignin and hemicelluloses, as well as related complexes in the substrate; (ii) chemical compounds, i.e., inhibitors and/or promoters; and (iii) pretreatment effects, i.e., the extent of lignin and xylan removal, lignin structure, pore volume, or accessible surface area [66]. The interaction between cellulosome and substrate-related factors is shown in Figure 3. The key parameters involved in the hydrolysis system may include two major functions (i.e., substrate-cellulosome interaction and cellulolysis reaction), eight mechanisms (grey circles), and their corresponding biological factors (white circles). Since it is nearly impossible to obtain a homogenous substrate that containing particles in the same size, it is difficult to get a pure cellulosomal enzyme expressed in the same level. All these substrate-related factors are interconnected and any single alteration would affect the others. The performance of pretreatment can directly affect the product yields and kinetics of the downstream processes, i.e., substrate hydrolysis. Some mechanisms of delignification for different pretreatment techniques have been confirmed while many questions still remain unanswered, which is mostly due to high complexity of lignin structure [67,68]. The substrate-related factors are the most sensitive and representative parameters include the substrate accessibility to cellulase [69], enzyme–additive interactions [70], crystallinity [71], and others [66,72].

### 3.1. Effects of Different Carbon Sources

Extracellular carbon sources affect the assembly of the cellulosome by regulating its enzymes and structural compositions, thus ensuring the present of optimal factors to break down the available carbohydrates [73,74,75]. Although approximately 90% of native or treated cellulosic substrates can be degraded by the appropriate cellulosomes, there are still many challenges for effective hydrolysis of different carbon sources by the cellulosome enzyme complexes [76,77].

Several previous studies initially demonstrated alterations of cellulosome compositions upon cultivation of the cellulosome producers on different carbon sources. Han et al. [78] cultured *C. cellulovorans* ATCC 35296 anaerobically in medium containing 1% (*w*/*v*) of Avicel, xylan, pectin and mixed polysaccharides (Avicel/xylan/pectin (3:1:1, by wt)) as carbon substrates, respectively. As a result, the cellulosome population was observed heterogeneously, although the scaffolding protein CbpA, endoglucanase EngE, and cellobiohydrolase ExgS were relatively constant. The cellulase activity was promoted by cellulosome contained CbpA, EngE/EngK, ExgS/EngH, and EngL in cells grew on a mixture of carbon sources, while high xylanase activity was detected in cellulosomes derived from cellulose, pectin and mixed carbon, which had larger amounts of XynB, XynA, and unknown proteins (35–45 kDa). These results indicated that the ratio of cellulosomal subpopulations in *C. cellulovorans* was controlled by its autogenous regulatory system that make up the cellulosomal population. 

Similarly, the cellulosome assembly of *C. thermocellum* strain DSM 1313 was examined for its response to available sole carbon sources, i.e., glucose, cellobiose, microcrystalline cellulose, alkaline-pretreated switchgrass, alkaline-pretreated corn stover, and dilute acid-pretreated corn stover [79]. Different catalytic and structural subunits (scaffoldins) were finally investigated in the different cellulosome samples. Cellulosomes derived from microcrystalline cellulose and glucose exhibited higher endoglucanase-to-exoglucanase ratios, as well as catalytic subunit-per-scaffoldin ratios compared to the lignocellulose-derived cellulosome types. The results verified glucose- and microcrystalline cellulose-derived cellulosomes were more efficient in their action on carbon sources than other cellulosome samples. Curiously, compared with the cellulosomes of strain *C. cellulovorans* ATCC 35296 grew on carbon sources such as Avicel, xylan, AXP (Avicel–xylan–pectin, 3:1:1), and cellobiose, the enzyme compositions between Avicel and cellobiose culture were similar and that almost no repression of cellulase enzymes when cells grew on cellobiose [63]. 

On the other hand, in order to understand the synergistic relationship between cellulosomes and noncellulosomal (hemi)cellulolytic enzymes, changes in mRNA and protein expression were examined with cultures of *C. cellulovorans* ATCC 35296 grew on cellobiose, cellulose, pectin, xylan, and corn fiber or mixtures, respectively [80]. Expression profiles of both the cellulosome and noncellulosomal enzymes were strongly affected by different carbon sources, whereas cellulosomal proteomes were more affected by the carbon source as compared to noncellulosomal enzymes. Furthermore, Fierobe et al. [81] compared hydrolysis effects of *C. cellulolyticum* cellulosome and free enzyme systems on recalcitrant substrates and tractable substrates. For the recalcitrant cellulose–Avicel, the presence of a CBM on scaffoldin and enzyme proximity on the organization of cellulosome chimeras contributed almost equally to the elevated action on the recalcitrant substrate, whereas the cellulosome chimeras exhibited little or no advantage over free enzymes on the tractable substrate–bacterial cellulose. 

Recently, cellulosomes displayed on the cell surface was compared between cells grew on soluble or recalcitrant insoluble substrates by using *C. clariflavum* [82]. According to immunolabeling of four cellulosome components: ScaA, ScaB, ScaC, and the most prominent enzyme, GH48, the results explored that the cellulosome producer required closely attached cellulosomes on its surface to break down the highly recalcitrant substrates. How these specific variations occur in response to the available carbon sources? One possibility is that the substrate-induced enzyme expressions determine the amounts of the various cellulosomal enzymes during the cellulosome assembly [77,80,83,84,85]. The other may cause by the specific interactions between the dockerins and their cognate cohesins [10,65,86]. Since certain cohesins can bind to enzymes by the docherins that are absent in other cohesins [10,15], the cellulosomal composition may varies with the enzyme expressions and the interactions of different cohesin–dockerin pairs. Moreover, Nataf et al. [87] revealed the cellulosomal regulatory mechanism at the genomic level. They suggested the cellulosomal genes were regulated via an extracellular sensing mechanism, in which alternative σ factors (i.e., σ^I1^ or σ^I6^) were activated in response to the carbohydrates in the extracellular surroundings.

It is known that the accumulation of carbon monomers such as glucose, cellobiose, as well as some other end products of hydrolysis will inhibit cellulases and decrease glucose yields [88,89,90]. In contrast to aerobic cellulase, kinetics studies related to cellulosome are quite limited owing to the intricate structure and catalytic mechanism. Lin et al. [91] described the utilization of recombinant anchoring cellulosome from *B. subtilis* W800N strain to degrade the *Chlorella* lipid-deprived residues. The kinetics parameters of maximum reaction rate (*V_max_*) and the Michaelis–Menten constant (*K_m_*) values displayed a prevailing effect when the cellulosome obtained in the supernatant as compared to the whole cells. Recently, a mathematical model was developed to estimate the inhibitory effect of glucose on cellulosome by using *C. thermocellum* (Equation (1)) [92]

(1) $$C=K-\frac{K\cdot A}{v}$$

where *C* was the glucose concentration, *K* was the inhibition constant for glucose on cellulosome, *v* was the rate of the hydrolytic action of cellulosome, and constant *A* could be deduced from the slope of the straight line, which plotted based on *C* versus 1/*v*. It described the relationship between glucose concentration and saccharification rate at a specific glucose concentration or a specific time. Glucose accumulation in a long term is independent to the saccharification rate at a specific time. Hence, methods that can decrease the glucose-induced inhibition on cellulosome should be effective in enhancing cellulose saccharification by the anaerobic cellulosome-producing bacteria [93]. Attempts to eliminate side reactions such as ethanol and CO_2_ fermentation proved the utilization of certain adsorbents (i.e., activated carbon and biochar) could lower the inhibition of glucose and improve the adsorption of substrates onto cellulosome [92].

### 3.2. Effects of Different Chemical Compounds

The CBP efficiency can potentially be improved by optimizing cellulosome activity and/or creating the synergy between cellulosomic saccharification and the subsequently fermentation. Various compounds generated by the pretreatment process or derived from the fermentation by-products usually are inhibitory to cell growth and fermentation activity [94,95,96]. These chemical compounds exist in the substrates significantly affect the cellulosome-induced biorefineries based on lignocellulosic biomass. Amongst these, the cellulosome activity of wild-type strain was inhibited by ethanol concentrations above 2% (*v*/*v*), whereas those evolved strains remained viable when ethanol concentrations increased up to 8% (*v*/*v*). Compared with commercial enzymes, *C. thermocellum* cellulosomes were generally able to tolerate higher ethanol concentrations [97]. In addition, in regard to the inhibitors released during the pretreatment of lignocellulosic biomass, typically, furfural and phenols are considered unfavorable side-products owing to their inhibitory effects on cell growth [98,99]. The *C. thermocellum* cellulosomes demonstrated tolerance on certain concentrations of furfural (≤5 mM), *p*-hydroxybenzoic acid (≤50 mM), and catechol (≤1 mM), respectively [97].

Parsiegla et al. [100] studied the chemical structure of the thiooligosaccharide methyl 4-S-β-cellobiosyl-4-thio-cellobioside (IG4), which performed as an inhibitor to the cellulosome of *C. cellulolyticum*. The orientation of the inhibitor molecules Inh1 and Inh2 was consistent with a processive action towards the non-reducing ends from the reducing ends of the cellulose chains. Moreover, You et al. [101] assembled a cellulosome-microbe complex ex vivo on the *Bacillus subtilis* surface, which displayed a mini-scaffoldin bound with three dockerin-containing cellulase components, i.e., endoglucanase Cel5, processive endoglucanase Cel9 and cellobiohydrolase Cel48. The hydrolysis performance indicated that high concentration cellodextrins in the boundary layer would inhibit cellulosome activity more strongly than short chain products because the β-glucosidase without a CBM usually works in the bulk phase [102]. Therefore, cellulosomes that expedite the cellulose bioconversion rate can help to construct CBP microorganisms with improved performance, which is expected to hydrolyze recalcitrant substrate efficiently under low secretory cellulase levels. 

On the other hand, the organic acids almost occur as products or by-products in microbial fermentation [103,104], in which both pH changes and anion accumulations occur in the bioreactor. The change in pH will drastically alter cellulosome capability for cellulose digestion [105,106,107]. In order to determine the effects of organic acid anions on cellulosome-induced cellulose hydrolysis, the cellulosomal enzyme activities of *C. thermocellum* JYT01 were investigated in the presence of formate, acetate, and lactate [97]. Interestingly, although these anions inhibited the cell growth, at the same time these acted as promoters to cellulosome activity at a concentration of formate, acetate, and lactate below 100, 200, and 50 mM, respectively, while negative effect was only observed beyond their critical concentrations. As a result, the promoted Avicel hydrolysis was achieved by supplementing exogenous organic acid anions in a living-cell culture. It presumed that the active domains of certain cellulosome harbor a moiety for specific anion-binding, and -promoting substrate recognitions in the presence of cellulose–anion compounds [97,108]. 

### 3.3. Effects of Pretreatment

Lignocellulosic biomass with only exterior surface is not applicable for the microbial digestion, owing to its low accessible surface areas. Pretreatment is considered crucial for valorization of lignocellulosic biomass into value-added bioproducts. The direct physical contact between the cellulosome producers and lignocellulosic surfaces are necessary to start the biocatalysis. Since pretreatment operations change several decisive factors concurrently, and it is hard to predict its effectiveness directly [66,109]. In fact, the effectiveness of pretreatment is usually evaluated by enzymatic hydrolysis or merely based on the yields of target products by fermentation [66]. Generally, the pretreatment process varies depending on the type of lignocellulosic biomass and there is no standalone method can be applied for all feedstock, because this varies with the type of natural biomass [110]. Currently, several available techniques have been developed to remove lignin from lignocellulosic biomass, i.e., acid pretreatment (such as organosolv or sulfite) and alkaline pretreatment (such as ammonia or NaOH). Table 1 compares the effect of different pretreatment on lignin structure and enzymatic hydrolysis. To the best of our knowledge, no special class of cellulases appear in cellulosomes because most of the cellulosomal enzymes belong to the same set of enzyme families as those of free cellulases. The understandings of free enzymes’ efficiency should provide a reliable foundation to evaluate the effects of pretreatment on cellulosomic catalysis.

Degradation of lignocellulosic biomass usually involves three steps: (i) enzyme adsorption to the substrate surface, (ii) hydrolysis of the substrate, and (iii) desorption of the cellulase into the liquid [129]. Similar to the microbial degradation, the lignocellulosic pore volume or accessible surface area for the cellulosomic enzymes is among the most affecting factors to the lignocellulose hydrolysis rate and yield. It means that once the diffusion of an enzyme molecule into a pore, the size of the enzymatic component should not be equal to the size of the pore owing to the wall confinement [130]. Moreover, when the pore size of lignocellulosic substrate is narrow, then β-glucosidase would not accompany other groups of cellulosomic cellulases into the pore. In other words, more spaces for synergistic actions between the different groups of cellulosomic cellulases are crucial for efficient hydrolysis of lignocellulosic biomass [66,130,131]. 

On the other hand, pretreatment is actually an important process to increase the surface area of substrate available for cellulosome. The total accessible surface area of lignocellulosic biomass is the sum of its external and internal surfaces, among which the external surface area depends on the size and shape of the material, while the internal surface area depends on its pore size and distribution [66,132]. Although higher than 90% of the sieved Avicel surface is accessible to free enzymes when it is in an average diameter of 100 μm [133], the large size of cellulosomes will prevent them from accessing many pores of the internal surface area. The presence of multiple enzymes on the cellulosomes can compensate for this limitation of cellulosomes to attack the binding sites in the pores [17,134]. Hence, the digestibility of lignocellulose for cellulosomes is significantly affected by the factor of accessible surface area, which will be gradually increased with the enzymatic hydrolysis caused by the removal of partial cellulose and hemicellulose. Besides the surface area, it is noticed that the cellulosomic hydrolysis rate also depends on the hydrolysis stages [79,135]. The rate of hydrolysis is normally rapid at the beginning stage and it becomes considerably slower during the latter stage, despite the availability of higher surface area. The slower hydrolysis rate should be a result of the higher crystallinity regions of the substrates, deactivation of the hydrolytic enzymes, and the increasing concentrations of the lignin [136,137].

In an attempt to increase the adsorption and hydrolysis rate of cellulosomal enzyme, Moraïs et al. [138] observed the effects of reduced recalcitrance on wheat straw degradation by using native and designer cellulosomes, respectively. Actions of cellulosomes were estimated either directly following the size reduction by mechanical treatment or an additional pretreatment by sodium hypochlorite to reduce the lignin content in order to promote enzymatic hydrolysis. The result without chemical pretreatment demonstrated that there was no significant effect on lignin content of the wheat straw substrate when utilized both the native and designer cellulosomes. Thus, although microbial enzymes demonstrate and ability to solubilize lignin and increase the cellulase access to cellulose [139,140], the cellulosomes hardly reduce cellulose content and/or decompose hemicellulose without prior pretreatment of the lignocellulosic substrate. However, it was reported that the chemical pretreatment of lignocellulose before enzymatic digestion usually generated lignocellulose-derived by-products such as phenolic compounds that would further inhibit the enzymatic saccharification [94,141]. To overcome this barrier, Davidi et al. [142] constructed a cellulosome with extra enzyme activities on lignin. The resultant chimera finally increased two-fold of the reducing sugars derived from wheat straw compared with the designed trivalent cellulosomes lacking the laccase, which can catalyze the oxidation of various phenolic and nonphenolic compounds [142,143].

## 4. Conclusions

Lignocellulosic biomass is a renewable resource with great potential to facilitate important bioproducts conversion by CBP in the context of biorefineries. However, the low rates and high costs of lignocellulose decomposition are the main barriers to commercialization of this biological conversion processes. To address these barriers, one significant area of heightened research activity is the study of naturally occurring cellulosomes produced by certain anaerobic bacteria. Numerous cellulosome-related investigations have been confirmed at the molecular level such as the structures and functions of cellulosomes, as well as gene modifications to enhance the biocatalysis of the enzyme complex, although the understanding of interactions between cellulosomes and their lignocellulosic substrate are still limited. 

In this review, the current understanding relating to several substrate-related physical and chemical factors affecting the activities of cellulosomes are summarized. Different carbon sources play significant impacts on the cellulosomal assembly by regulating the expression of enzyme activities and structural compositions. In addition, cellulosomic enzyme adsorption or desorption is an important biological parameter related to the degradation of the lignocellulosic substrates. Substrate accessibility is another crucial parameter of the lignocellulosic substrate, which is a desirable factor for all pretreatments. External surface area of the lignocellulosic biomass can be increased by the physical size reduction as well as changing of the particle shapes, while increase in the internal surface of the substrates should be followed by typical chemical or even biological pretreatments. Therefore, special attention should be paid to the pretreatment methods utilized for valorization of the lignocellulosic biomass into bioproducts prior to the cellulosomic catalysis. 

## Figures and Tables

**Table 1:** Alteration of lignin structure during pretreatment and their effects on enzymatic hydrolysis of pretreated substrate

Changes after Pretreatment	Effect of Pretreatment	Enzyme Efficiency	Feedstocks	Reference
Depletion of lignin content	Increase accessible surface area and porosity of substrate	Enhanced	Corn stover, sugarcane bagasse, *Eucalyptus globulus*	[111,112,113,114]
Formation of COOH	Reduce surface tension and increase electrostatic repulsion between lignin and enzymes	Enhanced	Aspen, corn stover, poplar, lodgepole pine	[115,116,117,118]
Sulphonation	Reduce surface tension and non-productive enzyme binding	Enhanced	Poplar, lodgepole pine, Norway spruce, black cottonwood	[118,119,120]
Alkoxylation of aliphatic side chains	Block lignin condensation	Enhanced	Beech	[121]
Reduced surface coverage by lignin	Increase porosity and surface area	Enhanced	Wheat straw, several wood and grass species	[122,123,124]
Formation of condensed units	Adsorb more enzymes due to hydrophobicity	Reduced	*Eucalyptus globulus*, red maple, loblolly pine, mixed hardwood, aspen, bamboo	[117,124,125,126]
Formation of phenolic OH	Hydrophobicity and hydrogen bonding	Reduced	Technical lignins, aspen, poplar, pine, bamboo, mixed hardwood, barley straw	[115,117,118,125]
Removal of aliphatic OH	Form more condense lignin	Reduced	*Eucalyptus globulus*, red maple, loblolly pine, mixed hardwood	[115,126]
Increased hydrophobicity	Adsorb more enzymes	Reduced	Poplar, lodgepole pine, bamboo	[118,125]
Formation of resinous products	Adsorb more enzymes	Reduced	Wheat straw	[127,128]

## References

[1] Yamakawa C.K., Qin F., Mussatto S.I. (2018). Advances and opportunities in biomass conversion technologies and biorefineries for the development of a bio-based economy. Biomass Bioenergy.

[2] Bauer F., Coenen L., Hansen T., McCormick K., Voytenko Palgan Y. (2017). Technological innovation systems for biorefineries: A review of the literature. Biofuels Bioprod. Biorefin..

[3] Periyasamy K., Santhalembi L., Mortha G., Aurousseau M., Boyer A., Subramanian S. (2018). Bioconversion of lignocellulosic biomass to fermentable sugars by immobilized magnetic cellulolytic enzyme cocktails. Langmuir.

[4] Amezcua-Allieri M.A., Sánchez-Duran T., Aburto J. (2017). Study of chemical and enzymatic hydrolysis of cellulosic material to obtain fermentable sugars. J. Chem..

[5] Chi X., Li J., Wang X., Zhang Y., Leu S., Wang Y. (2018). Bioaugmentation with *Clostridium tyrobutyricum* to improve butyric acid production through direct rice straw bioconversion. Bioresour. Technol..

[6] Olson D.G., McBride J.E., Shaw A.J., Lynd L.R. (2012). Recent progress in consolidated bioprocessing. Curr. Opin. Biotechnol..

[7] Wang Y., Tashiro Y., Sonomoto K. (2015). Fermentative production of lactic acid from renewable materials: Recent achievements, prospects, and limits. J. Biosci. Bioeng..

[8] Gilmore S.P., Henske J.K., O’Malley M.A. (2015). Driving biomass breakdown through engineered cellulosomes. Bioengineered.

[9] Arora R., Behera S., Kumar Sharma N., Kumar S. (2015). Bioprospecting thermostable cellulosomes for efficient biofuel production from lignocellulosic biomass. Bioresour. Bioprocess..

[10] Doi R.H., Kosugi A., Murashima K., Tamaru Y., Han S.O. (2003). Cellulosomes from mesophilic bacteria. J. Bacteriol..

[11] Lamed R., Setter E., Bayer E.A. (1983). Characterization of a cellulose-binding, cellulase-containing complex in *Clostridium thermocellum*. J. Bacteriol..

[12] Bayer E.A., Lamed R., White B.A., Flint H.J. (2008). From cellulosomes to cellulosomics. Chem. Rec..

[13] Desvaux M. (2005). *Clostridium cellulolyticum* model organism of mesophilic cellulolytic clostridia. FEMS Microbiol. Rev..

[14] Quiroz-Castañeda R.E., Folch-Mallol J.L., Chandel A. (2013). Hydrolysis of biomass mediated by cellulases for the production of sugars. Sustainable Degradation of Lignocellulosic Biomass Techniques, Applications and Commercialization.

[15] Artzi L., Bayer E.A., Moraïs S. (2017). Cellulosomes: Bacterial nanomachines for dismantling plant polysaccharides. Nat. Rev. Microbiol..

[16] Goncalves G.A.L., Mori Y., Kamiya N. (2014). Biomolecular assembly strategies to develop potential artificial cellulosomes. Sustain. Chem. Process..

[17] Moraïs S., Shterzer N., Lamed R., Bayer E.A., Mizrahi I. (2014). A combined cell-consortium approach for lignocellulose degradation by specialized *Lactobacillus plantarum* cells. Biotechnol. Biofuel..

[18] Béguin P., Lemaire M. (1996). The Cellulosome: An exocellular, multiprotein complex specialized in cellulose degradation. Crit. Rev. Biochem. Mol. Biol..

[19] Mazzoli R., Lamberti C., Pessione E. (2012). Engineering new metabolic capabilities in bacteria: Lessons from recombinant cellulolytic strategies. Trends Biotechnol..

[20] Pagès S., Bélaïch A., Fierobe H.P., Tardif C., Gaudin C., Bélaïch J.P. (1999). Sequence analysis of scaffolding protein CipC and ORFXp, a new cohesin-containing protein in *Clostridium cellulolyticum*: Comparison of various cohesin domains and subcellular localization of ORFXp. J. Bacteriol..

[21] Erickson S.J., Duvall S.W., Fuller J., Schrader R., MacLean P., Lowe J.R. (2013). Differential associations between maternal scaffolding and toddler emotion regulation in toddlers born preterm and full term. Early Hum. Dev..

[22] Bayer E.A., Bélaïch J.P., Shoham Y., Lamed R. (2004). The cellulosomes: Multienzyme machines for degradation of plant cell wall polysaccharides. Annu. Rev. Microbiol..

[23] Shoham Y., Lamed R., Bayer E.A. (1999). The cellulosome concept as an efficient microbial strategy for the degradation of insoluble polysaccharides. Trends Microbiol..

[24] Bomble Y.J., Beckham G.T., Matthews J.F., Nimlos M.R., Himmel M.E., Crowley M.F. (2011). Modeling the self-assembly of the cellulosome enzyme complex. J. Biol. Chem..

[25] Koukiekolo R., Cho H.Y., Kosugi A., Inui M., Yukawa H., Doi R.H. (2005). Degradation of corn fiber by *Clostridium cellulovorans* cellulases and hemicellulases and contribution of scaffolding protein CbpA. Appl. Environ. Microbiol..

[26] Fierobe H.P., Mingardon F., Mechaly A., Belaich A., Rincon M.T., Pages S., Lamed R., Tardif C., Belaich J.P., Bayer E.A. (2005). Action of designer cellulosomes on homogeneous versus complex substrates: Controlled incorporation of three distinct enzymes into a defined trifunctional scaffoldin. J. Biol. Chem..

[27] Valbuena A., Oroz J., Hervas R., Manuel Vera A., Rodriguez D., Menendez M., Sulkowska J.I., Cieplak M., Carrion-Vazquez M. (2009). On the remarkable mechanostability of scaffoldins and the mechanical clamp motif. Proc. Natl. Acad. Sci. USA.

[28] Gunnoo M., Cazade P.A., Galera-Prat A., Nash M.A., Czjzek M., Cieplak M., Alvarez B., Aguilar M., Karpol A., Gaub H. (2016). Nanoscale engineering of designer cellulosomes. Adv. Mater..

[29] Bras J.L.A., Alves V.D., Carvalho A.L., Najmudin S., Prates J.A.M., Ferreira L.M.A., Bolam D.N., Romao M.J., Gilbert H.J., Fontes C.M.G.A. (2012). Novel *Clostridium thermocellum* Type I cohesin–dockerin complexes reveal a single binding mode. J. Biol. Chem..

[30] Peer A., Smith S.P., Bayer E.A., Lamed R., Borovok I. (2009). Noncellulosomal cohesin- and dockerin-like modules in the three domains of life. FEMS Microbiol. Lett..

[31] Voronov-Goldman M., Yaniv O., Gul O., Yoffe H., Salama-Alber O., Slutzki M., Levy-Assaraf M., Jindou S., Shimon L.J.W., Borovok I. (2015). Standalone cohesin as a molecular shuttle in cellulosome assembly. FEBS Lett..

[32] Resch M.G., Donohoe B.S., Baker J.O., Decker S.R., Bayer E.A., Beckham G.T., Himmel M.E. (2013). Fungal cellulases and complexed cellulosomal enzymes exhibit synergistic mechanisms in cellulose deconstruction. Energ. Environ. Sci..

[33] Ichikawa S., Karita S., Kondo M., Goto M. (2011). Cellulosomal carbohydrate-binding module from *Clostridium josui* binds to crystalline and non-crystalline cellulose, and soluble polysaccharides. FEBS Lett..

[34] Goldstein M.A., Takagi M., Hashida S., Shoseyov O., Doi R.H., Segel I.H. (1993). Characterization of the cellulose-binding domain of the *Clostridium cellulovorans* cellulose-binding protein A (CbpA). J. Bacteriol..

[35] Tamaru Y., Karita S., Ibrahim A., Chan H., Doi R.H. (2000). A large gene cluster for the *Clostridium cellulovorans* cellulosome. J. Bacteriol..

[36] Caspi J., Irwin D., Lamed R., Li Y., Fierobe H.P., Wilson D.B., Bayer E.A. (2008). Conversion of *Thermobifida fusca* free exoglucanases into cellulosomal components: Comparative impact on cellulose-degrading activity. J. Biotechnol..

[37] Gerngross U.T., Romaniec M.P., Kobayashi T., Huskisson N.S., Demain A.L. (1993). Sequencing of a *Clostridium thermocellum* gene (cipA) encoding the cellulosomal SL-protein reveals an unusual degree of internal homology. Mol. Microbiol..

[38] Yaniv O., Jindou S., Frolow F., Lamed R., Bayer E.A. (2012). A simple method for determining specificity of carbohydrate-binding modules for purified and crude insoluble polysaccharide substrates. Methods Mol. Biol..

[39] Boraston A.B., Bolam D.N., Gilbert H.J., Davies G.J. (2004). Carbohydrate-binding modules: Fine-tuning polysaccharide recognition. Biochem. J..

[40] Shoseyov O., Shani Z., Levy I. (2006). Carbohydrate binding modules: Biochemical properties and novel applications. Microbiol. Mol. Biol. Rev..

[41] Hazlewood G.P., Romaniec M.P.M., Davidson K., Grépinet O., Béguin P., Millet J., Raynaud O., Aubert J.P. (1988). A catalogue of *Clostridium thermocellum* endoglucanase, β-glucosidase and xylanase genes cloned in *Escherichia coli*. FEMS Microbiol. Lett..

[42] Vazana Y., Moraïs S., Barak Y., Lamed R., Bayer E.A. (2010). Interplay between *Clostridium thermocellum* family 48 and family 9 cellulases in cellulosomal versus noncellulosomal states. Appl. Environ. Microbiol..

[43] Bayer E.A., Chanzy H., Lamed R., Shoham Y. (1998). Cellulose, cellulases and cellulosomes. Curr. Opin. Struct. Biol..

[44] Bayer E.A., Morag E., Lamed R. (1994). The cellulosome—A treasure-trove for biotechnology. Trends Biotechnol..

[45] Ding S.Y., Xu Q., Crowley M., Zeng Y., Nimlos M., Lamed R., Bayer E.A., Himmel M.E. (2008). A biophysical perspective on the cellulosome: New opportunities for biomass conversion. Curr. Opin. Biotechnol..

[46] Morag E., Halevy I., Bayer E.A., Lamed R. (1991). Isolation and properties of a major cellobiohydrolase from the cellulosome of *Clostridium thermocellum*. J. Bacteriol..

[47] Ravachol J., Borne R., Meynial-Salles I., Soucaille P., Pages S., Tardif C. (2015). Combining free and aggregated cellulolytic systems in the cellulosome-producing bacterium *Ruminiclostridium cellulolyticum*. Biotechnol. Biofuels.

[48] Morag E., Bayer E.A., Lamed R. (1993). Relationship of cellulosomal and noncellulosomal xylanases of *Clostridium thermocellum* to cellulose-degrading enzymes. J. Bacteriol..

[49] Mohand-Oussaid O., Payot S., Guedon E., Gelhaye E., Youyou A., Petitdemange H. (1999). The extracellular xylan degradative system in *Clostridium cellulolyticum* cultivated on xylan: Evidence for cell-free cellulosome production. J. Bacteriol..

[50] Maki M., Leung K.T., Qin W. (2009). The prospects of cellulase-producing bacteria for the bioconversion of lignocellulosic biomass. Int. J. Biol. Sci..

[51] Akinosho H., Yee K., Close D., Ragauskas A. (2014). The emergence of *Clostridium thermocellum* as a high utility candidate for consolidated bioprocessing applications. Front. Chem..

[52] Fan Z., Yuan L. (2010). Production of multifunctional chimaeric enzymes in plants: A promising approach for degrading plant cell wall from within. Plant Biotechnol. J..

[53] Cragg S.M., Beckham G.T., Bruce N.C., Bugg T.D., Distel D.L., Dupree P., Etxabe A.G., Goodell B.S., Jellison J., McGeehan J.E. (2015). Lignocellulose degradation mechanism sacross the tree of life. Curr. Opin. Chem. Biol..

[54] Chakraborty S., Fernandes V.O., Dias F.M.V., Prates JA.M., Ferreira L.M.A., Fontes C.M.G.A., Goyal A., Centeno M.S.J. (2015). Role of pectinolytic enzymes identified in *Clostridium thermocellum* cellulosome. PLoS ONE.

[55] Tamaru Y., Doi R.H. (2001). Pectate lyase A, an enzymatic subunit of the *Clostridium cellulovorans* cellulosome. Proc. Natl. Acad. Sci. USA.

[56] Perret S., Belaich A., Fierobe H.P., Belaich J.P., Tardif C. (2004). Towards designer cellulosomes in Clostridia: Mannanase enrichment of the cellulosomes produced by *Clostridium cellulolyticum*. J. Bacteriol..

[57] Sabathé F., Bélaïch A., Soucaille P. (2002). Characterization of the cellulolytic complex (cellulosome) of *Clostridium acetobutylicum*. FEMS Microbiol. Lett..

[58] Kurokawa J., Hemjinda E., Arai T., Karita S., Kimura T., Sakka K., Ohmiya K. (2001). Sequence of the *Clostridium thermocellum* mannanase gene man26B and characterization of the translated product. Biosci. Biotechnol. Biochem..

[59] Zverlov V.V., Fuchs K.P., Schwarz W.H. (2002). Chi18A, the endochitinase in the cellulosome of the thermophilic, cellulolytic bacterium *Clostridium thermocellum*. Appl. Environ. Microbiol..

[60] Tamaru Y., Miyake H., Kuroda K., Ueda M., Doi R.H. (2010). Comparative genomics of the mesophilic cellulosome-producing *Clostridium cellulovorans* and its application to biofuel production via consolidated bioprocessing. Envion. Technol..

[61] Vodovnik M., Logar R.M. (2010). Cellulosomes-promising supramolecular machines of anaerobic cellulolytic microorganisms. Acta. Chim. Slov..

[62] Gold N.D., Martin V.J.J. (2007). Global view of the *Clostridium thermocellum* cellulosome revealed by quantitative proteomic analysis. J. Bacteriol..

[63] Cho W., Jeon S.D., Shim H.J., Doi R.H., Han S.O. (2010). Cellulosomic profiling produced by *Clostridium cellulovorans* during growth on different carbon sources explored by the cohesin marker. J. Biotechnol..

[64] Tsai S.L., Goyal G., Chen W. (2010). Surface display of a functional minicellulosome by intracellular complementation using a synthetic yeast consortium and its application to cellulose hydrolysis and ethanol production. Appl. Environ. Microbiol..

[65] Artzi L., Morag E., Barak Y., Lamed R., Bayer E.A. (2015). *Clostridium clariflavum*: Key cellulosome players are revealed by proteomic analysis. mBio.

[66] Leu S.Y., Zhu J.Y. (2013). Substrate-related factors affecting enzymatic saccharification of lignocelluloses: Our recent understanding. Bioenergy Res..

[67] Chan K.L., Dong C., Wong M.S., Kim L.H., Leu S.Y. (2018). Plant chemistry associated dynamic modelling to enhance urban vegetation carbon sequestration potential via bioenergy harvesting. J. Clean. Prod..

[68] Sosnowski P., Wieczorek A., Ledakowicz S. (2003). Anaerobic co-digestion of sewage sludge and organic fraction of municipal solid wastes. Adv. Environ. Res..

[69] Carrère H., Dumas C., Battimelli A., Batstone D.J., Delgenès J.P., Steyer J.P., Ferrer I. (2010). Pretreatment methods to improve sludge anaerobic degradability: A review. J. Hazard. Mater..

[70] Zhen G., Lu X., Kato H., Zhao Y., Li Y.Y. (2017). Overview of pretreatment strategies for enhancing sewage sludge disintegration and subsequent anaerobic digestion: Current advances, full-scale application and future perspectives. Renew. Sustain. Energy Rev..

[71] Liu H., Sun J., Leu S.Y., Chen S. (2016). Toward a fundamental understanding of cellulase-lignin interactions in the whole slurry enzymatic saccharification process. Biofuels Bioprod. Biorefin..

[72] Pan X., Gilkes N., Kadla J., Pye K., Saka S., Gregg D., Ehara K., Xie D., Lam D., Saddler J. (2006). Bioconversion of hybrid poplar to ethanol and co-products using an organosolv fractionation process: Optimization of process yields. Biotechnol. Bioeng..

[73] Bhat S., Goodenough P.W., Owen E., Bhat M.K. (1993). Cellobiose: A true inducer of cellulosome in different strains of *Clostridium thermocellum*. FEMS Microbiol. Lett..

[74] Bae J., Morisaka H., Kuroda K., Ueda M. (2013). Cellulosome complexes: Natural biocatalysts as arming microcompartments of enzymes. J. Mol. Microbiol. Biotechnol..

[75] Han S.O., Yukawa H., Inui M., Doi R.H. (2003). Transcription of *Clostridium cellulovorans* cellulosomal cellulase and hemicellulose genes. J. Bacteriol..

[76] Raman B., Pan C., Hurst G.B., Rodriguez M.J., McKeown C.K., Lankford P.K., Samatova N.F., Mielenz J.R. (2009). Impact of pretreated switchgrass and biomass carbohydrates on *Clostridium thermocellum* ATCC 27405 cellulosome composition: A quantitative proteomic analysis. PLoS ONE.

[77] Morisaka H., Matsui K., Tatsukami Y., Kuroda K., Miyake H., Tamaru Y., Ueda M. (2012). Profile of native cellulosomal proteins of *Clostridium cellulovorans* adapted to various carbon sources. AMB Express.

[78] Han S.O., Yukawa H., Inui M., Doi R.H. (2005). Effect of carbon source on the cellulosomal subpopulations of *Clostridium cellulovorans*. Microbiology.

[79] Yoav S., Barak Y., Shamshoum M., Borovok I., Lamed R., Dassa B., Hadar Y., Morag E., Bayer E.A. (2017). How does cellulosome composition influence deconstruction of lignocellulosic substrates in *Clostridium* (Ruminiclostridium) thermocellum DSM 1313?. Biotechnol. Biofuels.

[80] Han S.O., Cho H.Y., Yukawa H., Inui M., Doi R.H. (2004). Regulation of expression of cellulosomes and noncellulosomal (hemi)cellulolytic enzymes in *Clostridium cellulovorans* during growth on different carbon sources. J. Bacteriol..

[81] Fierobe H.B., Bayer E.A., Tardif C., Czjzek M., Mechaly A., Belaich A., Lamed R., Shoham Y., Belaich J.P. (2002). Degradation of cellulose substrates by cellulosome chimeras. Substrate targeting versus proximity of enzyme components. J. Biol. Chem..

[82] Artzi L., Dadosh T., Milrot E., Moraïs S., Levin-Zaidman S., Morag E., Bayer E.A. (2018). Colocalization and disposition of cellulosomes in *Clostridium clariflavum* as revealed by correlative superresolution imaging. mBio.

[83] Han S.O., Yukawa H., Inui M., Doi R.H. (2003). Regulation of expression of cellulosomal cellulase and hemicellulase genes in *Clostridium cellulovorans*. J. Bacteriol..

[84] Kosugi A., Murashima K., Doi R.H. (2001). Characterization of xylanolytic enzymes in *Clostridium cellulovorans*: Expression of xylanase activity dependent on growth substrates. J. Bacteriol..

[85] Murashima K., Kosugi A., Doi R.H. (2002). Determination of subunit composition of *Clostridium cellulovorans* cellulosomes that degrade plant cell walls. Appl. Environ. Microbiol..

[86] Park J.S., Matano Y., Doi R.H. (2001). Cohesin–dockerin interactions of cellulosomal subunits of *Clostridium cellulovorans*. J. Bacteriol..

[87] Nataf Y., Bahari L., Kahei-Raifer H., Borovok I., Lamed R., Bayer E.A., Sonenshein A.L., Shoham Y. (2010). *Clostridium thermocellum* cellulosomal genes are regulated by extracytoplasmic polysaccharides via alternative sigma factors. Proc. Natl. Acad. Sci. USA.

[88] Qing Q., Yang B., Wyman C.E. (2010). Xylooligomers are strong inhibitors of cellulose hydrolysis by enzymes. Bioresour. Technol..

[89] Teugjas H., Valjamae P. (2013). Product inhibition of cellulases studied with ^14^C-labeled cellulose substrates. Biotechnol. Biofuels.

[90] Carere C.R., Sparling R., Cicek N., Levin D.B. (2008). Third generation biofuels via direct cellulose fermentation. Int. J. Mol. Sci..

[91] Lin C.C., Kan S.C., Yeh C.W., Chen C.I., Shieh C.J., Liu Y.C. (2015). Kinetics Study for the Recombinant Cellulosome to the Degradation of Chlorella Cell Residuals.

[92] Zhang P., Wang B., Xiao Q., Wu S. (2015). A kinetics modeling study on the inhibition of glucose on cellulosome of *Clostridium thermocellum*. Bioresour. Technol..

[93] Lu Y., Zhang Y.H.P., Lynd L.R. (2006). Enzyme–microbe synergy during cellulose hydrolysis by *Clostridium thermocellum*. Proc. Natl. Acad. Sci. USA.

[94] Dong C., Wang Y., Zhang H., Leu S.Y. (2018). Feasibility of high-concentration cellulosic bioethanol production from undetoxified whole Monterey pine slurry. Bioresour. Technol..

[95] Weil J.R., Dien B., Bothast R., Hendrickson R., Mosier N.S., Ladisch M.R. (2002). Removal of fermentation inhibitors formed during pretreatment of biomass by polymeric adsorbents. Ind. Eng. Chem. Res..

[96] Min S., Kim O.J., Bae J., Chung T.N. (2018). Effect of pretreatment with the NADPH xxidase inhibitor Apocynin on the therapeutic efficacy of human placenta-derived mesenchymal stem cells in intracerebral hemorrhage. Intl. J. Mol. Sci..

[97] Xu C., Qin Y., Li Y., Ji Y., Huang J., Song H., Xu J. (2010). Factors influencing cellulosome activity in consolidated bioprocessing of cellulosic ethanol. Bioresour. Technol..

[98] Duarte L., Carvalheiro F., Neves I., Girio F. (2005). Effects of aliphatic acids, furfural, and phenolic compounds on *Debaryomyces hansenii* CCMI 941. Appl. Biochem. Biotechnol..

[99] Guarnieri M.T., Franden M.A., Johnson C.W., Beckham G.T. (2017). Conversion and assimilation of furfural and 5-(hydroxymethyl)furfural by *Pseudomonas putida* KT2440. Metabol. Eng. Commun..

[100] Parsiegla G., Juy M., Reverbel-Leroy C., Tardif C., Velaich J.P., Driguez H., Haser R. (1998). The crystal structure of the processive endocellulase CelF of *Clostridium cellulolyticum* in complex with a thiooligosaccharide inhibitor at 2.0 Å resolution. EMBO J..

[101] You C., Zhang X.Z., Sathitsukssanoh N., Lynd L.R., Zhang Y.H.P. (2012). Enhanced microbial utilization of recalcitrant cellulose by an ex vivo cellulosome-microbe complex. Appl. Environ. Microbiol..

[102] Zhang Y.H.P. (2011). Substrate channeling and enzyme complexes for biotechnological applications. Biotechnol. Adv..

[103] Alonso S., Rendueles M., Diaz M. (2013). Microbial production of specialty organic acids from renewable and waste materials. Crit. Rev. Biotechol..

[104] Wackett L.P. (2018). Microbial acid fermentation products: An annotated selection of world wide web sites relevant to the topics in microbial biotechnology. Microb. Biotechnol..

[105] Zverlov V.V., Velikodvorskaya G.A., Schwarz W.H. (2003). Two new cellulosome components encoded downstream of celI in the genome of *Clostridium thermocellum*: The non-processive endoglucanase CelN and the possibly structural protein CseP. Microbiology.

[106] Bras J.L., Pinheiro B.A., Cameron K., Cuskin F., Viegas A., Najmudin S., Bule P., Pires V.M.R., Romao M.J., Bayer E.A. (2016). Diverse specificity of cellulosome attachment to the bacterial cell surface. Sci. Rep..

[107] Leis B., Held C., Andreeen B., Liebl W., Graubner S., Schulte L.P., Schwarz W.H., Zverlov V.V. (2018). Optimizing the composition of a synthetic cellulosome complex for the hydrolysis of softwood pulp: Identification of the enzymatic core functions and biochemical complex characterization. Biotechnol. Biofuels.

[108] Correia M.A., Prates J.A., Bras J., Fontes C.M., Newman J.A., Lewis R.J., Gilbert H.J., Flint J.E. (2008). Crystal structure of a cellulosomal family 3 carbohydrate esterase from *Clostridium thermocellum* provides insights into the mechanism of substrate recognition. J. Mol. Biol..

[109] Taherzadeh M., Karimi K. (2008). Pretreatment of lignocellulosic wastes to improve ethanol and biogas production: A review. Int. J. Mol. Sci..

[110] Zabed H., Sahu J.N., Boyce A.N., Faruq C. (2016). Fuel ethanol production from lignocellulosic biomass: An overview on feedstocks and technological approaches. Renew. Sustain. Energy Rev..

[111] Ding S.Y., Liu Y.S., Zeng Y., Himmel M.E., Baker J.O., Bayer E.A. (2012). How does plant cell wall nanoscale architecture correlate with enzymatic digestibility?. Science.

[112] Siqueira G., Várnai A., Ferraz A., Milagres A.M.F. (2013). Enhancement of cellulose hydrolysis in sugarcane bagasse by the selective removal of lignin with sodium chlorite. Appl. Energy.

[113] Wallace J., Brienzo M., García-Aparicio M.P., Görgens J.F. (2016). Lignin enrichment and enzyme deactivation as the root cause of enzymatic hydrolysis slowdown of steam pretreated sugarcane bagasse. New Biotechnol..

[114] Zhang L., Zhang L., Zhou T., Wu Y., Xu F. (2016). The dual effects of lignin content on enzymatic hydrolysis using film composed of cellulose and lignin as a structure model. Bioresour. Technol..

[115] Guo F., Shi W., Sun W., Li X., Wang F., Zhao J., Qu Y. (2014). Differences in the adsorption of enzymes onto lignins from diverse types of lignocellulosic biomass and the underlying mechanism. Biotechnol. Biofuels.

[116] Nakagame S., Chandra R.P., Kadla J.F., Saddler J.N. (2011). Enhancing the enzymatic hydrolysis of lignocellulosic biomass by increasing the carboxylic acid content of the associated lignin. Biotechnol. Bioeng..

[117] Sun S., Huang Y., Sun R., Tu M. (2016). The strong association of condensed phenolic moieties in isolated lignins with their inhibition of enzymatic hydrolysis. Green Chem..

[118] Yang Q., Pan X. (2016). Correlation between lignin physicochemical properties and inhibition to enzymatic hydrolysis of cellulose. Biotechnol. Bioeng..

[119] Li M., Pu Y., Ragauskas A.J. (2016). Current understanding of the correlation of lignin structure with biomass recalcitrance. Front. Chem..

[120] Pareek N., Gillgren T., Jönsson L.J. (2013). Adsorption of proteins involved in hydrolysis of lignocellulose on lignins and hemicelluloses. Bioresour. Technol..

[121] Lancefield C.S., Panovic I., Deuss P.J., Bartac K., Westwood N.J. (2017). Pre-treatment of lignocellulosic feedstocks using biorenewable alcohols: Towards complete biomass valorisation. Green Chem..

[122] Kim J.S., Lee Y.Y., Kim T.H. (2016). A review on alkaline pretreatment technology for bioconversion of lignocellulosic biomass. Bioresour. Technol..

[123] Sipponen M.H., Pihlajaniemi V., Pastinen O., Laakso S. (2014). Reduction of surface area of lignin improves enzymatic hydrolysis of cellulose from hydrothermally pretreated wheat straw. RSC Adv..

[124] Sipponen M.H., Rahikainen J., Leskinen T., Pihlajaniemi V., Mattinen M.L., Lange H., Crestini C., Österberg Ö.M. (2017). Structural changes of lignin in biorefinery pretreatments and consequences to enzyme-lignin interactions. Nord. Pulp Pap. Res. J..

[125] Huang C., He J., Min D., Lai C., Yong Q. (2016). Understanding the nonproductive enzyme adsorption and physicochemical properties of residual lignins in moso bamboo pretreated with sulfuric acid and kraft pulping. Appl. Biochem. Biotechnol..

[126] Yu Z., Gwak K.S., Treasure T., Jameel H., Chang H.M., Park S. (2014). Effect of lignin chemistry on the enzymatic hydrolysis of woody biomass. ChemSusChem.

[127] Kumar R., Hu F., Sannigrahi P., Jung S., Ragauskas A.J., Wyman C.E. (2013). Carbohydrate derived-pseudo-lignin can retard cellulose biological conversion. Biotechnol. Bioeng..

[128] Rasmussen H., Tanner D., Sørensen H.R., Meyer A.S. (2017). New degradation compounds from lignocellulosic biomass pretreatment: Routes for formation of potent oligophenolic enzyme inhibitors. Green Chem..

[129] Taherzadeh M.J., Karimi K. (2007). Enzyme-based hydrolysis processes for ethanol from lignocellulosic materials: A review. Bioresources.

[130] Bubner P., Dohr J., Plank H., Mayrhofer C., Nidetzky B. (2012). Cellulases dig deep: In Situ observation of the mesoscopic structural dynamics of enzymatic cellulose degradation. J. Biol. Chem..

[131] Karimi K., Taherzadeh M.J. (2016). A critical review on analysis in pretreatment of lignocelluloses: Degree of polymerization, adsorption/desorption, and accessibility. Bioresour. Technol..

[132] Shafiei M., Kumar R., Karimi K., Karimi K. (2015). Pretreatment of lignocellulosic biomass. Lignocellulose-Based Bioproducts.

[133] Neuman R.P., Walker L.P. (1992). Solute exclusion from cellulose in packed columns: Experimental investigation and pore volume measurements. Biotechnol. Bioeng..

[134] Xu Q., Resch M.G., Podkaminer K., Yang S., Baker J.O., Donohoe B.S., Wilson C., Klingeman D.M., Olson D.G., Decker S.R. (2016). Dramatic performance of *Clostridium thermocellum* explained by its wide range of cellulase modalities. Sci. Adv..

[135] Wilson D.B. (2010). Demonstration of the importance for cellulose hydrolysis of CelS, the most abundant cellulosomal cellulase in *Clostridium thermocellum*. Proc. Natl. Acad. Sci. USA.

[136] Wang J., Gao G., Li Y., Yang L., Liang Y., Jin H., Han W., Feng Y., Zhang Z., Wang J. (2015). Cloning, expression, and characterization of a thermophilic endoglucanase, AcCel12B from *Acidothermus cellulolyticus* 11B. Int. J. Mol. Sci..

[137] Khodaverdi M., Jeihanipour A., Karimi K., Taherzadeh M.J. (2012). Kinetic modeling of rapid enzymatic hydrolysis of crystalline cellulose after pretreatment by NMMO. J. Ind. Microbiol. Biotechnol..

[138] Moraïs S., Morag E., Barak Y., Goldman D., Hadar Y., Lamed R., Shoham Y., Wilson D.B., Bayer E.A. (2012). Deconstruction of lignocellulose into soluble sugars by native and designer cellulosomes. mBio.

[139] Keller F.A., Hamilton J.E., Nguyen Q.A. (2003). Microbial pretreatment of biomass: Potential for reducing severity of thermochemical biomass pretreatment. Appl. Biochem. Biotechnol..

[140] Shi J., Sharma-Shivappa R., Chinn M., Howell N. (2009). Effect of microbial pretreatment on enzymatic hydrolysis and fermentation of cotton stalks for ethanol production. Biomass Bioenergy.

[141] Jönsson L.J., Martín C. (2016). Pretreatment of lignocellulose: Formation of inhibitory byproducts and strategies for minimizing their effects. Bioresour. Technol..

[142] Davidi L., Moraïs S., Artzi L., Knop D., Hadar Y., Arfi Y., Bayer E.A. (2016). Toward combined delignification and saccharification of wheat straw by a laccase-containing designer cellulosome. Proc. Natl. Acad. Sci. USA.

[143] Glazunova O.A., Moiseenko K.V., Kamenihina I.A., Isaykina T.U., Yaropolov A.I., Fedorova T.V. (2019). Laccases with variable properties from different strains of *Steccherinum ochraceum*: Does glycosylation matter?. Int. J. Mol. Sci..

